# Supplementary material for: Identification of photoperiod-induced specific miRNAs in the adrenal glands of Sunite sheep (Ovis aries)
Source: Front Vet Sci. 2022 Jul 22;9:888207. doi: 10.3389/fvets.2022.888207 (PMC9354845; doi:10.3389/fvets.2022.888207)
Supplement: Supplementary file 1 [file Data_Sheet_1.ZIP › Supplementary file/miRDeep2 file.zip!/miRDeep2 file/Know_Structure/LP42A1/oar-let-7f.pdf]

miRBase precursor : oar-let-7f
Total read count : 701620
oar-let-7f read count : 701480
remaining reads : 140

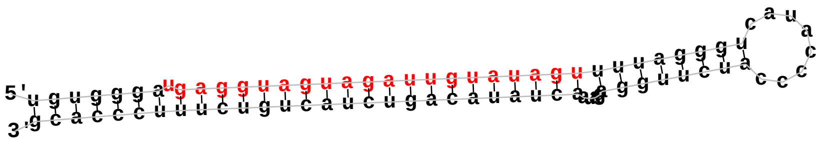

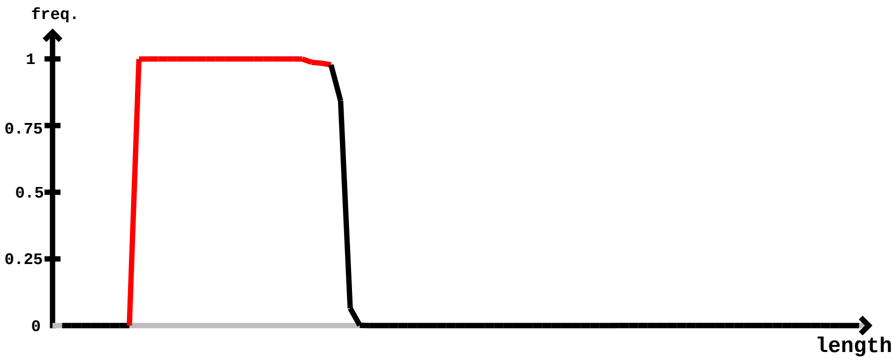


```
         oar-let-7f
5'- uguggga ugagguaguagauuguauagu uuuagggucauaccccaucuuggagauaacuauacagucuacugucuuucccacg   -3'    exp
    .((((((.((((((((((((((((((((((((((((.........))))))))....)))))))))))))))))))))))))).   reads   mm    sample
    ......augagguaguagauuguau...........................................................   3       0     seq
    ......augagguaguagauuguauag.........................................................   2       0     seq
    ......augagguaguagauuguauagu........................................................   13      0     seq
    ......augagguaguagauuguauaguu.......................................................   32      0     seq
    ......Gugagguaguagauuguauaguu.......................................................   6       1     seq
    ......augagguaguagauuguauaguuu......................................................   3       0     seq
    .......ugagAuaguagauugua............................................................   2       1     seq
    .......ugagguaguagauuguU............................................................   8       1     seq
    .......ugagguaguaAauugua............................................................   3       1     seq
    .......ugagguaguagauugAa............................................................   1       1     seq
    .......ugaAguaguagauugua............................................................   1       1     seq
    .......ugagguaguagauugua............................................................   227     0     seq
    .......ugagguaguagauuguau...........................................................   8679    0     seq
    .......ugagguaguUgauuguau...........................................................   10      1     seq
    .......ugagguaguagauuguUu...........................................................   9       1     seq
    .......ugaAguaguagauuguau...........................................................   69      1     seq
    .......ugagguaguagauuguaua..........................................................   2171    0     seq
    .......ugagguaguagauugAaua..........................................................   1       1     seq
    .......ugagguaguagauugAauag.........................................................   1       1     seq
    .......ugagguaguagauuguauag.........................................................   4269    0     seq
    .......ugagguaguagauugAauagu........................................................   16      1     seq
    .......ugagguaguagauuguauagu........................................................   94666   0     seq
    .......ugagguaguagauuguauaguu.......................................................   545299  0     seq
    .......ugagguaguagauuguauaguuu......................................................   42834   0     seq
    .......ugagguaguagauuguauaguuG......................................................   2084    1     seq
    .......ugagguaguagauuguauaguuuu.....................................................   477     0     seq
    .......ugagguaguagauuguauaguuGu.....................................................   133     1     seq
    .......ugagguaguagauuguauaguuuua....................................................   6       0     seq
    ........Uagguaguagauuguau...........................................................   1       1     seq
    ........gagguaguagauuguau...........................................................   2       0     seq
    ........gagguaguagauuguaua..........................................................   3       0     seq
    ........gagguaguagauuguauag.........................................................   4       0     seq
    ........gagguaguagauuguauagu........................................................   52      0     seq
    ........gagguaguagauuguauaguu.......................................................   310     0     seq
    ........gagguaguagauuguauaguuG......................................................   2       1     seq
```

oar-let-7f

```
uguggga ugagguaguagauuguauagu uuuagggucauaccccaucuuggagauaacuauacagucuacugucuuucccacg
```

```
ugugggaugagguaguagauuguauaguuuuagggucauaccccaucuuggagauaacuauacagucuacugucuuucccacg
........gagguaguagauuguauaguuu.....................................................    24    0    seq
.........agguaguagauuguauagu.......................................................    3     0    seq
.........agguaguagauuguauaguu......................................................    5     0    seq
.........agguaguagauuguauaguuG.....................................................    2     1    seq
.........agguaguagauuguauaguuGu....................................................    38    1    seq
..........gguaguagauuguauagu.......................................................    1     0    seq
..........gguaguagauuguauaguu......................................................    2     0    seq
..........gguaguagauuguauaguuu.....................................................    1     0    seq
...........guaguagauuguauaguu......................................................    2     0    seq
............uaguagauuguauaguu......................................................    3     0    seq
.............................uuagggucauaccccaucuuggag..............................    1     0    seq
.........................................................cuauacagucuacugucu........    1     0    seq
.........................................................cuauacagucuacugucuuu......    6     0    seq
.........................................................cuauacagucuacugucuuuc.....    25    0    seq
.........................................................cuauacagucuacugucuuucc....    107   0    seq
```
